# CDK1 serves as a novel therapeutic target for endometrioid endometrial cancer

**DOI:** 10.7150/jca.51139

**Published:** 2021-02-22

**Authors:** Xue Ying, Xuan Che, Jianzhang Wang, Gen Zou, Qin Yu, Xinmei Zhang

**Affiliations:** 1 Women's Hospital, School of Medicine, Zhejiang University, Hangzhou, Zhejiang, P.R. China, 310006.; 2 Jiaxing University Affiliated Women and Children Hospital, Jiaxing, Zhejiang, P.R. China, 314000.

**Keywords:** endometrial cancer, CDK1, RO3306, targeted therapy, bioinformatics analysis

## Abstract

**Background:** Endometrial cancer (EC) is one of the most common and prevalent gynecologic malignancies worldwide. The aim of this study was to identify a novel therapeutic target for endometrioid endometrial cancer.

**Materials and Methods:** Bioinformatic analysis was performed and CDK1 was screen out as one of the hub genes in the pathogenesis of EC. Immunohistochemistry was used to verify the expression of CDK1 in endometrial cancer tissue. Cell viability and colony formation were used to study the effects of CDK1 on the proliferation and colony formation of endometrial cancer cells *in vitro*. Apoptosis and cell cycle assays were used to elucidate the mechanism of CDK1 affecting cell proliferation. Tumor xenograft transplantation assay was performed to show the effects of CDK1 on the growth of endometrial cancer cells *in vivo*.

**Results:** CDK1 was over expressed in endometrioid endometrial cancer, and accumulation of cytoplasmic CDK1 was associated with histological grade of EC. CDK1 promoted endometrial cancer cell growth and colony formation *in vitro*. The inhibition of CDK1 activity induced cell apoptosis and caused G2/M phase arrest of cell cycle in endometrial cancer cells. The inhibition of CDK1 activity also inhibited endometrial cancer growth in xenograft models.

**Conclusion:** CDK1 was involved in the pathogenesis of endometrioid endometrial cancer and provided a novel therapeutic target for endometrioid endometrial cancer.

## Introduction

Endometrial cancer (EC) is one of the most common and prevalent gynecologic malignancies worldwide, with 382069 new cases and 89929 deaths predicted in 2018. The mobility and mortality of EC are on the rise globally 1-3. Surgical therapy has improved over time, which has not reduced mortality rates. Current non-surgical treatment options for EC are limited, and new management strategies are therefore needed 4, especially for those who do not want to lose fertility. A categorization of endometrial cancer subtypes was proposed by Bokhman in 1983 5. That system proposed 2 broad types of endometrial cancer. Histologically, type I tumors are predominantly well differentiated to moderately differentiated endometrioid tumors while type II tumors are mainly nonendometrioid tumor types. Instead of treating all the different histological types as one disease, modern clinical trials are targeting biologic subsets 6. Here, our study focused on stage I or II endometrioid endometrial cancer (G1 or G2).

Similar to other cancers, the development of EC must be understood as a multistep process that accumulates genetic and epigenetic changs. Many studies have been devoted to understanding mutations in oncogenes and tumor suppressor genes during the progression of this tumor. Mutations in these genes, such as PTEN, KRAS, CTNNB1, and PIK3CA, are thought to be associated with the pathogenesis of endometrioid endometrial cancer 7, 8.

CDK1 was screen out to be one of the hub genes in the development of endometrial cancer by our study. CDK1, with a molecular weight of 34 kD, is encoded by cell division cycle gene 2 (cdc2) and belongs to the Ser/Thr protein kinase family 9, 10. CDK1, as a key determinant of mitosis progression, can initiate mitosis 11, and the key event of CDK1 is the control of G2/M 12. The direct impact and potential mechanisms of CDK1 on cell cycle regulation had been extensively investigated and clearly elucidated by many lectures 13, 14. Cyclin-dependent protein kinases (CDKs) are important proteins that are essential for the expression and regulation of numerous components which are necessary for the cell cycle regulation 13. In normal cells, CDKs play an important role in the regulation of cell cycle, and the aberrant expression of CDKs allows cancer cells to escape the normal regulation of cell cycle 15. There are 20 CDKs (1-20) in human 16. The CDKs family was firstly discovered as the cell cycle regulating proteins (CDKs 1-6, 11, 14-18) 17, which can be divided into the interphase CDKs (CDK2, CDK4, and CDK5), mitotic CDK1 and transcriptional CDKs (CDK7 and CDK9) according to their function in the cell cycle regulation 15. Loss of CDKs eventually leads to the cellular apoptosis 15. CDKs are natural targets for anticancer therapy because of their role in cell proliferation 16. FDA approved palbociclib, ribociclib, and abemaciclib as CDK4/6 inhibitors for the treatment of breast cancer 16. A few studies claimed that the loss of interphase CDKs has no significant effect on cell cycle regulation, because CDK1 can fully compensate for the absence of these CDKs 18. CDK1 was the only nonredundant cell cycle regulator in CDK family 19.

Previous studies revealed that many factors such as progestin, miRNA, adipocyte and urolithin A, inhibited the tumorigenicity of endometrial cancer cells by targeting CDK1 20-27. CDK1 was a key mediator for them to play the anticancer role. The direct role of CDK1 on the tumorigenicity of endometrium was not well illustrated. The expression and function of CDK1 in EC were investigated by our study. We found that CDK1 was over expressed in endometrioid endometrial cancer and the expression of CDK1 in cytoplasm increased as tumor grade progressed. *In vitro* and *in vivo*, CDK1 could promote the growth and proliferation of endometrial cancer cells. These results suggested that CDK1 was associated with the pathogenesis of EC and was a putative target for the treatment of endometrioid endometrial cancer.

## Materials and methods

### Patient sample collection and RNA extraction

The study protocol was approved by the Human Ethics Committee of the Women's Hospital (2019ER-R NO.039), School of Medicine, Zhejiang University. Seven patients who received hysterectomy and diagnosed with stage I or II endometrioid endometrial carcinoma (G1 or G2) according to the 1988 International Federation of Gynecology and Obstetrics (FIGO) criteria were enrolled at Women's Hospital, Medical school of Zhejiang University in 2018 28. At the same time, six patients received diagnostic curettage and diagnosed with benign endometrium were enrolled as the control group. None of the patients received hormone therapy, chemotherapy or radiation therapy within 3 months before surgery, and none had a history of cancer. Written informed consent was obtained from every patient.

Fresh tissue samples were immediately frozen in liquid nitrogen and stored at -80 °C. Samples were sent to Genergy Biotechnology Corporation (http://www.genenergy.cn/) for RNA extraction. According to the manufacturer's instructions, a sequencing library was created with the use of the TruSeq RNA sample prep kit (Illumina). Then Illumina HiSeq X Ten was used to sequence the library as 151-bp paired-end reads.

### PPI network construction and hub genes identification

Differentially Expressed Genes (DEGs) between endometrioid endometrial cancer and normal endometrium were screened out using |log2FC| ≥ 2 and P value ≤ 0.05 as cut-off criterion and the TOP 25% of DEGs were selected for the PPI analysis. The interactions between these proteins were analyzed using The Search Tool for the Retrieval of Interacting Genes (STRING, http://www.string-db.org/). Cytoscape3.6.1 was used to visualize the network and calculate node degree, node betweenness, and closeness. The higher scores of these 3 indicators, the more central the node is in the network 29, 30. Degree was used to screen hub genes of PPI network in our study.

### Endometrial cancer specimens and Immunohistochemistry (IHC) assay

A total of 63 cases were enrolled for IHC assay. 33 endometrioid endometrial cancer tissues and 30 benign endometrium tissues were obtained from Women's Hospital (Zhejiang University School of Medicine). Clinical and pathological data of all patients were retrieved from their records at our hospital. The 33 endometrial cancers patients were all diagnosed with stage I or II endometrioid endometrial carcinoma (G1 or G2). The 30 normal controls were all received hysterectomy for benign disease such as myoma and pathological assay conferred benign endometrium. None received hormone therapy, chemotherapy or radiation therapy within 3 months. None has a history of cancer. The patients were staged according to the 1988 International Federation of Gynecology and Obstetrics (FIGO) criteria 28. Pathological diagnoses of endometrial tissues were classified and graded by pathologists according to the 1994 World Health Organization criteria. The use of the tissues and the collection of information about the patients were all approved by the Human Ethics Committee of the Women's Hospital (2019ER-R NO.039), School of Medicine, Zhejiang University.

Sections of 4 μM thick were immunostained using mouse monoclonal antibody against CDK1 (dilution1:100; Santa Cruz Biotechnology, CA, USA), following the procedure reported by our team previously 31. The immunohistochemical scores were also utilized based on the previous study 31.

### Human endometrial cancer cells culture

Human endometrial cancer cell line (HEC-1-B) was obtained from Chinese Academy of Sciences Cell Bank and then cultured in Dulbecco's modified Eagle's medium (DMEM) (Gibco, NewYork, USA). The medium was supplemented with 10% fetal bovine serum (FBS) (Sigma, CA, USA), 50 U/ml penicillin, and 50 μg/ml streptomycin at 37 °C in a humidified atmosphere containing 5% CO_2_.

### Cell viability assay

Cell proliferation ability was measured using the Cell Counting Kit-8 (CCK-8) following the manufacturer's instruction (Dojindo Molecular Technologies Inc, Dojindo, JPN). CDK1 inhibitor RO3306 (Selleck Chemicals, Houston, USA) was dissolved in DMSO at a concentration of 20 mM and added to the cell culture medium at final concentrations of 0, 2.5, 5, 10, 15 and 20 μM. HEC-1-B cells (1×10^4^ cells/well) were cultured in a 96-well plate in culture medium with different concentration of RO3306 for 24, 48 or 72 h. Following culture, the medium was removed and 100 μl fresh medium containing Kit-8 reagents was added to each well for 2 h and optical density (OD) was detected at a wavelength of 490 nm using an enzyme-labeled analyzer. The optical density values were measured at least three times against reagent blank. Cell viability was determined by the formula: (A_treated_/A_control_) *100%.

### Colony formation assay

The HEC-1-B cells were planted in a 12-well (1×10^3^ cells/well) plate in medium with different concentration of RO3306 (0, 2.5 and 5 μM) for 48 h. During colony growth, the culture medium was replaced every 3 days for 10 days. After fixing with 10% formaldehyde for 20 min and staining with 0.5% crystal violet for 30 min, colonies with diameter greater than 0.1 mm were scored.

### Apoptosis Assay

Annexin V-FITC/PI double staining kit (Beyotime, Shanghai, China) was used to assess apoptosis. HEC-1-B cells (1×10^6^ cells/well) were cultured in a 6-well plate in culture medium with different concentrations (0, 5 and 10 μM) of RO3306 for 24 or 48 h. Cells were collected and stained following the instructions of manufacturer. The level of cell apoptosis was quantified by Flow cytometry.

### Cell cycle analysis

Propidium iodide (BectonDickinson, CA, USA) was used for cell cycle analysis. HEC-1-B cells (1×10^6^ cells/well) were cultured in a 6-well plate in culture medium with different concentration (0, 5 and 10 μM) of RO3306 for 24 or 48 h. Cells were collected and fixed following the instructions of manufacturer. The cell cycle was analyzed by BD FACSVerse.

### Tumor xenograft transplantation assay

Xenograft assay was used to explore whether RO3306 can affect the growth of endometrial cancer cells *in vivo*. Female BALB/c nude mice (4-6 weeks old and 15-20 g) were purchased from Shanghai Animal Center, Chinese Academy of Science (Shanghai, China). After 1 week of acclimation, each mouse was subcutaneously injected with HEC-1-B cells (2×10^6^) on the left flank. When most of the tumor volume was greater than 40 mm^3^, the mice were divided into the RO3306 treatment group and the control group blindly and randomly. There was no significant difference in tumor volume and body weight between the two groups. The mice in the RO3306 treatment group were intragastrically administered with RO3306 4mg/kg every 2 days. The control group were treated with vehicle (DMSO). Tumor volume and body weight were measured every 4 days. The tumor volume was calculated by the formula V=L×W^2^/2, where L is the length in millimeters and W is the width in millimeters. The total treatment lasted for 28 days, and the mice were harvested at the end of the treatment. The weight of the grafts was measured.

All animal studies were conducted in accordance with the regulations of the Chinese government on animal care and the protocol approved by the Ethics Committee of Animal Research of Zhejiang University (16206).

### Statistical Analysis

Data was expressed as mean ± SD. Statistical analysis was performed using IBM SPSS 20.0 statistical software program (Chicago, IL, USA). Student's independent or paired t-tests were used for continuous data. Constituent ratio data were evaluated with chi-square (χ^2^) tests. Statistical difference was considered to be significant at a value of *p* <0.05 (*), highly significant at a value of *p* <0.01 (**) and extremely significant when *p* <0.001 (***) or *p* <0.0001 (****).

## Results

### CDK1 was screened out as one of hub genes using the PPI network analysis

**Figure 1:**
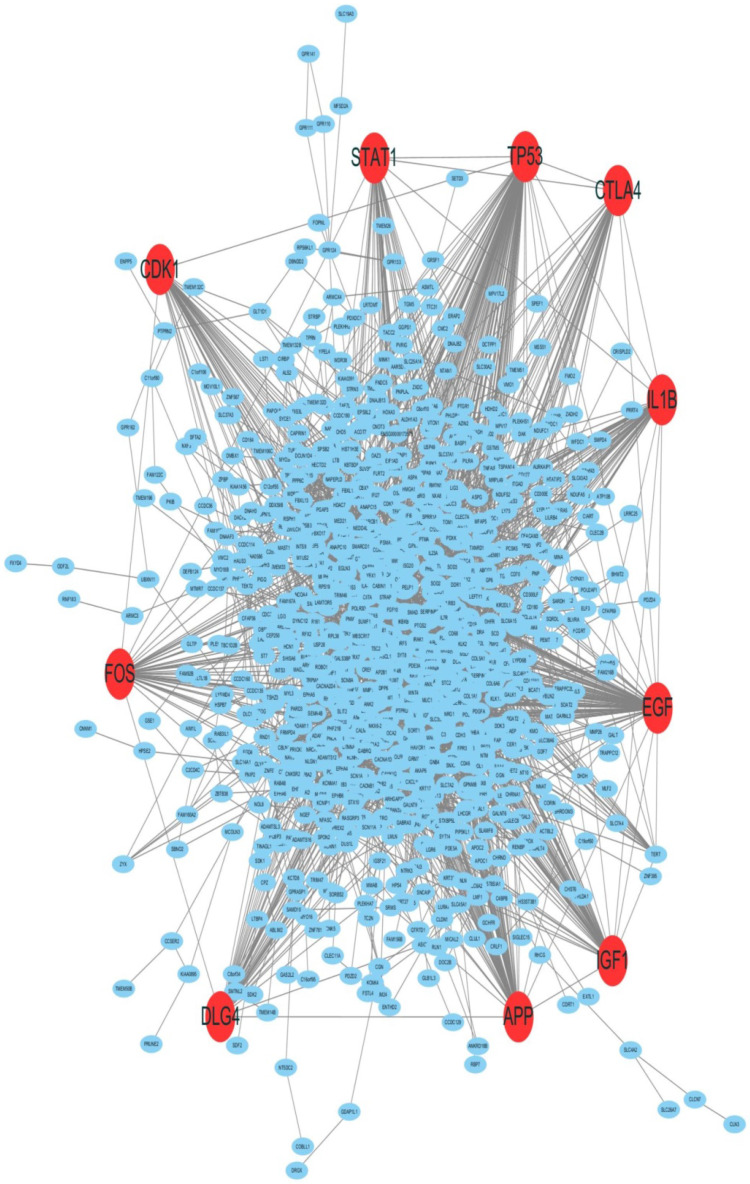
The PPI network of DEGs between endometrioid endometrial cancer and normal endometrium. 10 hub genes were screened out using PPI analysis. The red nodes represented the 10 hub genes. CDK1 was one of the hub genes.

By employing differential gene expression analysis between endometriold endometrial cancer and normal endometrium, 4227 DEGs were screened out, in which 2768 DEGs were up-regulated and 1459 DEGs were down-regulated by using |log2FC| ≥ 2 and P value ≤ 0.05 as cut-off criterion. The Top 25% of significantly up-regulated and down-regulated DEGs were selected for PPI analysis. The PPI network for the DEGs between EC and normal endometrium was shown in Figure 1. The PPI network was constructed with 963 nodes and 4048 edges. Among the 963 nodes, 10 hub genes (red in Figure 1) were identified with the filtering of degree >45 criteria, and the 10 hub genes were TP53, EGF, APP, IGF1, IL1B, DLG4, FOS, CTLA4, CDK1 and STAT1. The degree, node betweenness and closeness of the ten hub genes were shown in Table 1. The functions of the 10 hub genes were investigated through literature review and CDK1 was selected for further investigation.

### CDK1 was over expressed in human endometrioid endometrial cancer and accumulation of cytoplasmic CDK1 is associated with histological grade of endometrioid endometrial cancer

**Figure 2:**
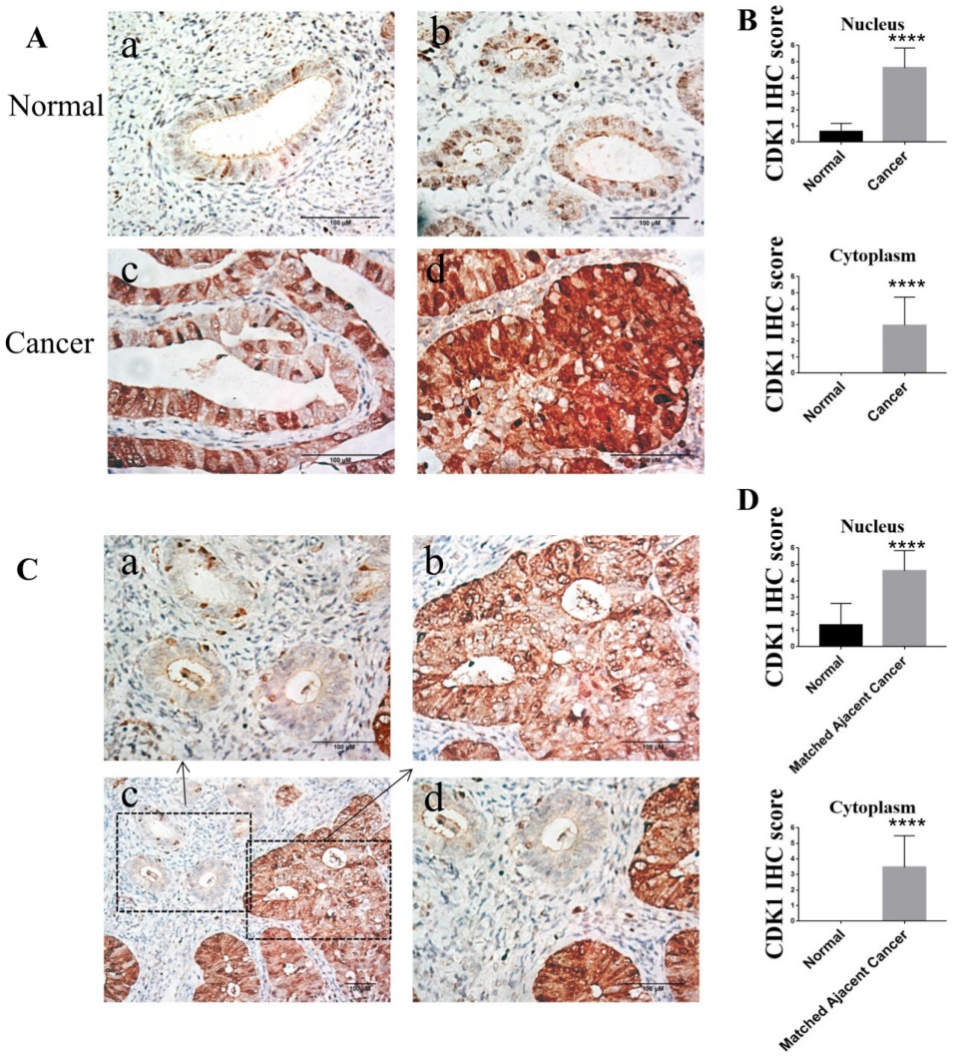
The expression of CDK1 in normal endometrium tissues and endometrioid endometrial cancer tissues. (A) Representative different types of immunostaining for CDK1 in normal endometrium tissues and endometrioid endometrial cancer tissues (original magnification×400). (a) CDK1-negative nuclear and cytoplasmic staining in the majority of normal endometrium tissues. (b) CDK1-positive nuclear staining but CDK1-negative cytoplasmic staining in the minority of normal endometrium tissues. (c) CDK1 strong positive nuclear staining but CDK1 weak positive cytoplasmic staining in the well differentiated endometrioid endometrial cancer tissues. (d) Diffuse strong positive staining for CDK1 in moderately differentiated endometrioid endometrial cancer tissues. (B) The average IHC scores in the nucleus and cytoplasm of normal endometrium tissues (n = 30) were compared with the scores in the nucleus and cytoplasm of cancer tissues (n = 33). Student's independent t-tests was used. Data were presented as means ± SD, ****p <0.0001. (C) Representative immunohistochemical staining for CDK1 in normal endometrium tissues and its matched adjacent cancer tissues. Representative immunostaining for CDK1 in normal endometrium tissues (a) and its matched adjacent cancer tissues (b) of endometrioid endometrial cancer (original magnification×400). (c) A geographic zone of normal endometrium tissues (upper and left) contrasting with matched adjacent region of cancer tissues (down and right half) (original magnification×200). (d) Higher power view of the central region of panel C (original magnification×400). Scale bars 100 µm. (D) The average IHC scores in the nucleus and cytoplasm of normal endometrium tissues (n = 33) were compared with the scores in the nucleus and cytoplasm of matched adjacent cancer tissues (n = 33). Student's paired t-tests was used. Data were presented as means ± SD, ****p <0.0001.

To compare the expression of CDK1 protein in human endometrioid endometrial cancer and normal endometrium, immunohistochemistry of 33 endometrioid endometrial cancer tissue samples and 30 normal endometrium tissue samples were performed. Compared to normal endometrium, the expression of CDK1 in human endometrioid endometrial cancer tissues was significantly elevated, accumulated in both nucleus and cytoplasm (*p* < 0.0001) (Figure 2A-B and Table 2). The percentage of positive expression of cytoplasmic CDK1 in endometrial cancer (57.6%) was significantly higher than that in normal endometrium (0.0%), while the percentage of positive expression of nuclear CDK1 in endometrial cancer (97.0%) was significantly higher than that in normal endometrium (3.3%) (Table 3). In addition, CDK1 expression in endometrial cancer tissues was also significantly higher than that in matched adjacent normal endometrium tissues (*p* < 0.0001) (Figure 2C-D). The expression of CDK1 in different tissues could be summered as follow: nuclear CDK1 expression in cancer tissue > cytoplasmic CDK1 expression in cancer tissue > nuclear CDK1 expression in normal tissue > cytoplasmic CDK1 expression in normal tissue (Figure 2).

To determine whether CDK1 protein elevation was related to clinicopathologic characteristics, the relationship of CDK1 expression and clinicopathologic characteristics was analyzed. The cytoplasmic CDK1 expression increased as tumor grade progressed (*p* <0.001) (Table 4). The cytoplasmic CDK1 expression in Grade 1 (well differentiated) endometrial cancer tissues was lower than that in Grade 2 (moderately differentiated) endometrial cancer tissues (Figure 2A c-d).

Therefore, the elevation of CDK1 expression in the nucleus and cytoplasm was important for the pathogenesis of endometriold endometrial cancer. In addition, cytoplasmic CDK1 expression is associated with histological grade (the degree of differentiation) of endometrial cancer.

### CDK1 promoted endometrial cancer cell growth and colony formation *in vitro*

**Figure 3:**
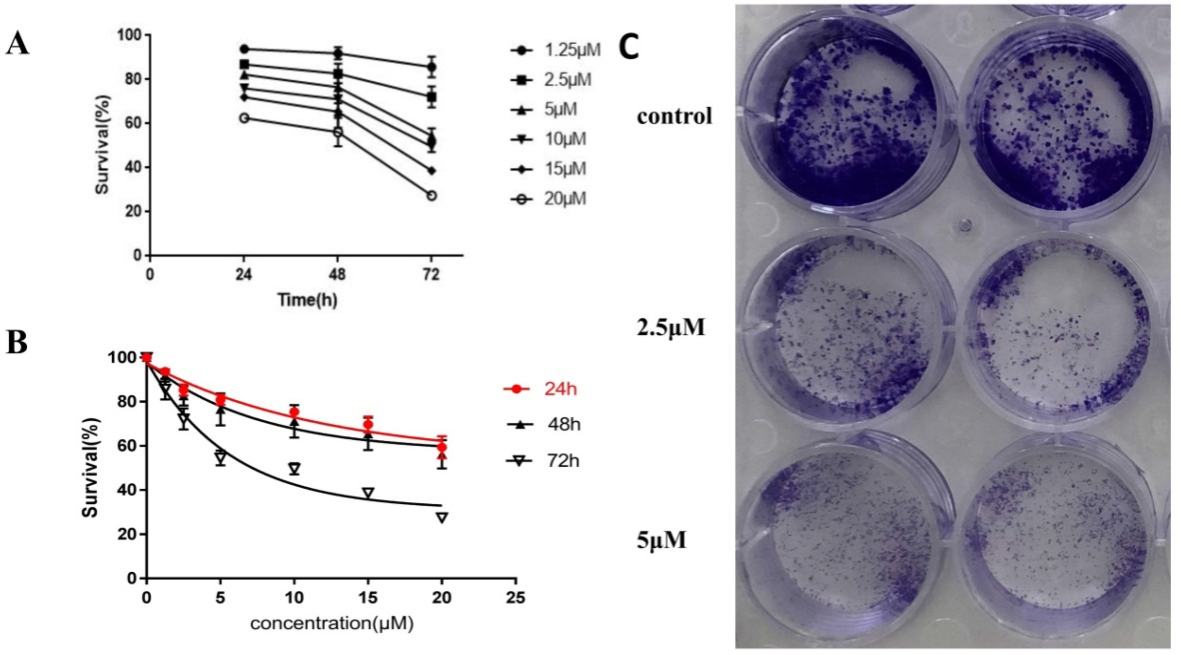
RO3306 as a potent CDK1 inhibitor inhibited the cell growth and colony formation of HEC-1-B cells *in vitro*. (A, B) HEC-1-B cells were treated with RO3306 at different concentrations of 0, 2.5, 5, 10, 15 and 20 µM. Cell viability was determined at 24, 48, and 72 hours by a CCK-8 assay. Cell viability values are expressed relative to those for cells without drug exposure. Data were presented as means ± SD (n = 3). (A) Time-response effect of RO3306 on HEC-1-B cells. (B) Dose-response effect of RO3306 on HEC-1-B cells. The IC50 value for RO3306 at 72 h were obtained by GraphPad Prism version 7.0 software. (C) Colony formation assay. HEC-1-B cells were treated with RO3306 at different concentrations of 0, 2.5 and 5 µM for 48 h. Cells were fixed and stained at the end of the study.

To detect the effect of CDK1 on the growth and proliferation of endometrial cancer cells, RO3306 (a potent CDK1 inhibitor) was used to inhibit the activity of CDK1. The human endometrial cancer cell line HEC-1-B, one of the commonly used tumor cell lines as models for studying human endometrial cancer, was applied in our experiments. The time and dose response of RO3306 on the proliferation of HEC-1-B was measured *in vitro* by a CCK8 assay. RO3306 showed a time-dependent and dose-dependent inhibition of the growth of HEC-1-B cells (Figure 3A-B). The half-maximal inhibitory concentration (IC50) value of RO3306 at 72h was 7.87 µM for HEC-1-B cells. These findings indicated that RO3306 potently inhibited the growth and proliferation of HEC-1-B cells in a time and dose dependent manner. Besides, to further verify the inhibition of RO3306 on HEC-1-B cell proliferation, a plate colony formation assay was carried out. RO3306 significantly suppressed the clone formation of HEC-1-B cells (Figure 3C). These data revealed that CDK1 played a critical role in the growth and proliferation of HEC-1-B cells while RO3306 could inhibit its roles.

### RO3306 induced cell apoptosis and caused G2/M phase arrest of cell cycle in endometrial cancer cells

**Figure 4:**
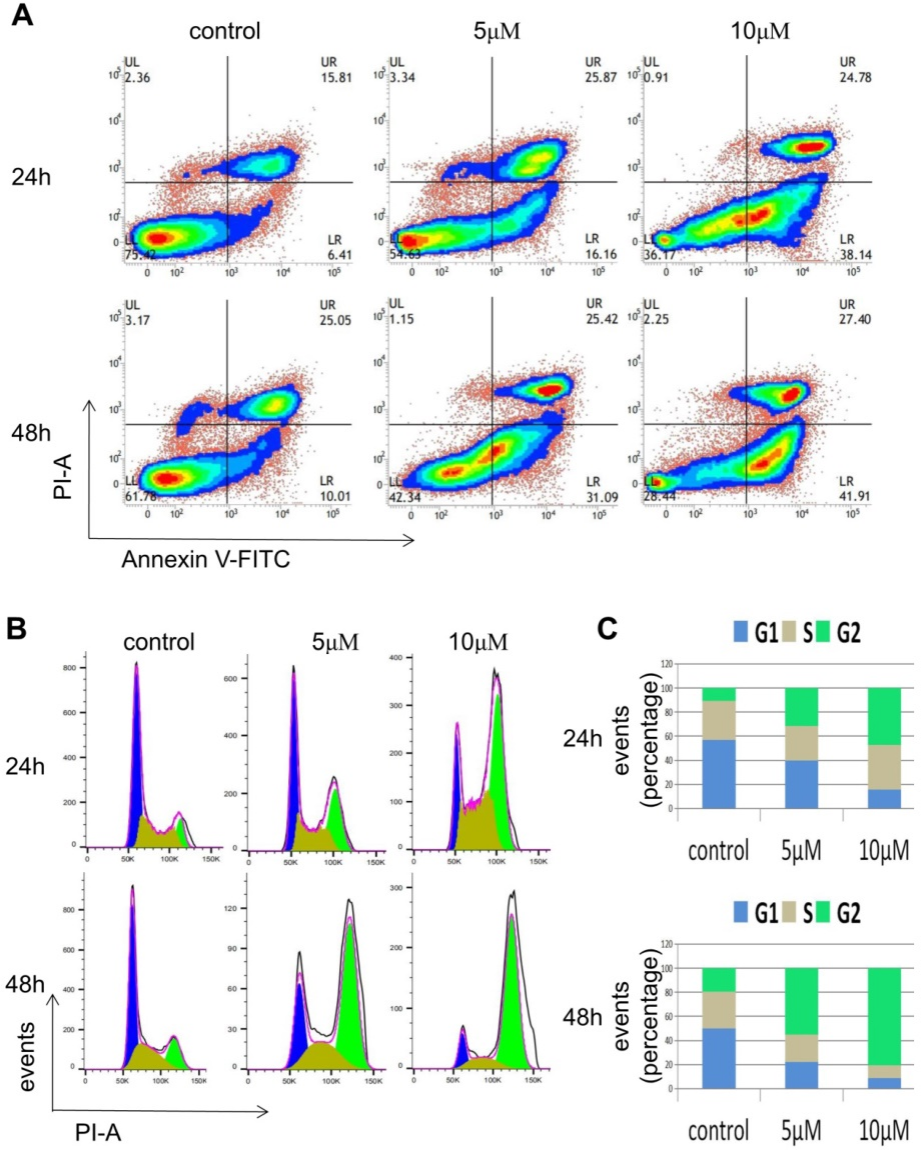
RO3306 inhibited HEC-1-B cell growth through induction of apoptosis and G2/M phase arrest. (A) HEC-1-B cells were treated with RO3306 at different concentrations of 0, 5 and 10 µM for 24 or 48 h. Apoptosis assay was performed with Annexin V-FITC/PI double staining using FACS analysis. (B) HEC-1-B cells were treated with RO3306 at different concentrations of 0, 5 and 10 µM for 24 or 48 h. Cell cycle assay was performed with PI staining using FACS analysis. The representative images of 3 independent experiments were shown. (C) Quantification of B. Results are the means (n = 3).

To investigate how the inhibition effect of RO3306 on the growth of HEC-1-B cells was caused, FACS analysis was carried out. HEC-1-B cells were treated with RO3306 at different concentrations (0, 5, 10 M) and for various time periods (24 or 48 h). In the apoptosis assay, the proportion of early apoptotic cells of HEC-1-B increased from 6.4% to 16.16% after treatment with 5 μM RO3306 for 24 h, while the proportion increased from 6.4 to 38.14% with 10 μM RO3306 for 24 h. Similarly, the proportion of early apoptotic cells increased from 10.01 to 31.09% after treatment with 5 μM RO3306 for 48 h, and the proportion increased from 10.01 to 41.91% after treatment with 10 μM RO3306 for 48 h. Thus, RO3306 had apoptosis-inducing effect on HEC-1-B cells (Figure 4A).

In the cell cycle analysis, the proportion of HEC-1-B cells in G2 phase increased from 10.8% to 30.4% after treatment with 5 μM RO3306 for 24 h, while the proportion increased from 10.8% to 46.3% after treatment with 10 μM RO3306 for 24 h. Similarly, the proportion increased from 19% to 55.9% after treatment with 5μM RO3306 for 48 h, while the proportion increased from 19% to 81.7% after treatment with 10 μM RO3306 for 48 h (Figure 4B-C). With the treatment of RO3306, the proportion of cells in G2 phase was significantly increased and the proportion of cells in G1 phase and S phase was significantly decreased, indicating that CDK1 played a key role in the G2/M transition of the cell cycle.

These results suggested that RO3306 might exert the inhibition of cell proliferation through both inducing cell apoptosis and affecting G2/M phase of the cell cycle progress.

### RO3306 inhibited endometrial cancer growth in xenograft models

**Figure 5:**
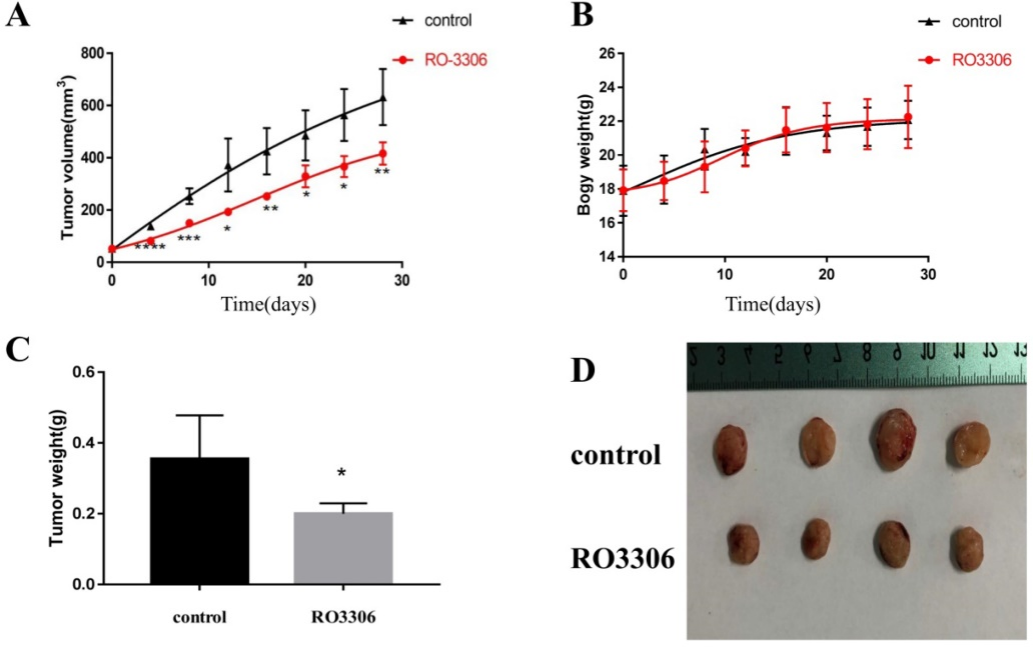
RO3306 inhibited the growth of HEC-1-B cells *in vivo*. (A) The increment of xenograft tumor volume over time in xenograft models of HEC-1-B cells with two different treatments (vehicle or RO3306). Drugs were oral gavage administered once every 2 days and DMSO was served as vehicle control. The data shown represent mean ± SD from four samples (*p <0.05; **p <0.01; ***p <0.001; ****p <0.0001). (B) The changes of body weight of mice in two groups (the control group and the RO3306 treatment group). The data shown represent mean ± SD from four samples (p >0.05). (C) The tumor weights of the two groups (the control group and the RO3306 treatment group) at the end of the study (*p < 0.05). (D) Gross images of xenograft tumor masses of the two groups (the control group and the RO3306 treatment group) at the end of the study.

Considering that RO3306 reduced the growth and proliferation of HEC-1-B cells *in vitro* (Figure 3), we subsequently evaluated the effect of RO3306 on the growth of HEC-1-B cells *in vivo*. Subcutaneous tumor xenografts developed from HEC-1-B cells in BALB/c nude mice were successfully established individually. Consistent with the *in vitro* results, the tumor growth of xenograft endometrial cancer grafts was significantly blocked with the treatment of RO3306 (Figure 5A-D).

As early as day 4 of the treatment, there was a extremely significant difference in tumor volume between the RO3306 treatment group and the control group (*p* <0.0001) (Figure 5A). At the end of the study, tumor volumes of endometrial cancer grafts in the RO3306 treatment group and the control group were 392.2 ± 24.34 and 689.8 ± 104.3mm^3^, respectively, with highly significant differences between the two groups (*p* <0.01) (Figure 5A). As to body weight, there was no significant difference between the two groups (*p* >0.05) (Figure 5B). The tumor weight of endometrial cancer grafts harvested at the end of the study in the RO3306 treatment group was significantly lower than that in the control group (*p* <0.05) (Figure 5C). These data suggested that CDK1 played an important role in the growth and proliferation of endometrial cancer cells *in vivo* and RO3306 can serve as its targeted inhibitor for the treatment of endometrioid endometrial cancer.

## Discussion

Using bioinformatics screens of hub genes, we found that CDK1 was one of the hub genes in the development of EC. Other researchers also found the same result as we did. CDK1 was found to be one of the hub genes in the development of endometrial cancer using different databases 32-35. CDK1 was also found to be the hub gene in other kinds of cancers, such as cervical cancer and lung cancer 36-38.

We found CDK1 was over expressed in human endometrioid endometrial cancer tissues. Similar to our finding, CDK1 was found to be over expressed in human endometrial cancer tissues by other researches 39-41. Furthermore, CDK1 was over expressed and had values on estimating prognosis of different kinds of cancers, such as ovarian cancer, breast cancer, hepatocellular cancer, gastric cancer and lung cancer 42-49. Another interesting finding of our study was that the accumulation of cytoplasmic CDK1 was associated with histological grade of endometrioid endometrial cancer. Cytoplasmic CDK1 is more frequently expressed higher in G2 endometrial endometrial cancer than that in G1 endometrial endometrial cancer. In consistent with our finding, cytoplasmic Cdk1 increased with progression of histological grade of epithelial ovarian cancer accordingly 50. Different from our findings, Zhang C found loss of cytoplasmic CDK1 predicts poor survival in human lung cancer 51. Sun W found the high ratio of nuclear and cytoplasmic expression of Cdk1 expression was meaningful to predict poor prognosis of colorectal cancer 52. The cytoplasmic expression and function of CDK1 was not clear and controversial, which deserved further investigation.

An important finding by our study was that CDK1 promoted endometrial cancer cell growth and colony formation *in vitro*. The knockdown of CDK1 by siRNA or the inactivation of CDK1 by CDK1 inhibitors achieved the same result that the growth of ovarian cancer cancers was inhibited 44, 50. Also, CDK1 inhibitor could restrict the growth of liver cancer stem cells 46.

With the development of selective CDK1 inhibitor, more investigation about CDK1 as a target gene for molecular therapy is promising 16. RO3306 is a potent and selective inhibitor of CDK1 and can serve as an ideal compound to investigate the function of CDK1 19. RO3306 was found to induce cell apoptosis and cause G2/M phase arrest of cell cycle in endometrial cancer cells by our study. Similar to our founding RO3306 could induce G2/M cell cycle arrest in colorectal cancer cells, colon cancer cells, cervical cancer cells, ovarian cancer cells, hepatocellular cancer cells and MYC-dependent human breast cancer cells 19, 46, 50, 53. We found RO3306 induced endometrial cancer cell apoptosis by FACS analysis. Wookyeom Yang found similar results using epithelial ovarian cancer cells. As to the levels of apoptotic marker proteins, cleaved PARP and cleaved caspase-3, Wookyeom Yang did not found consistent results using different ovarian cancer cell lines. The CDK1 related apoptotic pathway was not so clear and we planned to investigate it in future.

RO3306 effectively blocked tumor growth of EC grafts *in vivo*. Consistent with our finding, RO3306 as a single drug inhibited tumor growth in two other animal cancer models, including hepatocellular cancer and epithelial ovarian cancer 46, 50. In combined therapy studies, RO3306 had synergistic antitumor activity with sorafenib or cisplatin 46, 50. With the development of targeted therapy, the synergistic anti-tumor activity of targeted therapy drugs and traditional chemotherapy drugs are very promising 54, 55 and need further investigation.

There were several limitations of our study. Firstly, only the human endometrial cancer cell line (HEC-1-B) was studied by our study. We will repeat the experiments using primary endometrial cancer cells in future. Another limitation of our study was that the CDK1 related apoptosis signal pathways were not investigated yet and need further investigation. The third limitation of our study was lack of an independent cohort study. In this study, we mainly focus on the basic research using cell line and mouse model, and our result may supply an experimental foundation for the further clinical study of CDK1 inhibitor. Therefore, we will do an independent cohort study to further validate the main findings and conclusions in the future.

## Conclusions

In summary, we first found that CDK1 was one of the hub genes in the pathogenesis of EC. We revealed that CDK1 was over expressed in EC. Inhibition of the activity of CDK1 could inhibit the growth of endometrial cancer cells *in vitro* and *in vivo*. Together, CDK1 could serve as a novel therapeutic target for EC patients.

## Figures and Tables

**Table 1:** The degree, betweenness and closeness of the 10 hub genes

name	Degree	Betweenness Centrality	Closeness Centrality
TP53	133	0.206	0.432
EGF	90	0.081	0.407
APP	84	0.063	0.398
IGF1	62	0.045	0.390
IL1B	56	0.039	0.373
DLG4	49	0.039	0.356
FOS	47	0.032	0.384
CTLA4	47	0.028	0.363
CDK1	46	0.040	0.367
STAT1	46	0.028	0.365

**Table 2:** CDK1 IHC scores in nucleus and cytoplasm of normal endometrium and endometrial cancer

	Scores in nucleus				Scores in cytoplasm			
	Mean±SD	95% CI	Range	*p* value	Mean±SD	95% CI	Range	*p* value
Normal (N=30)	0.67±0.49	(0.49-0.85)	0-2.4		0.00±0.00	(0-0)	0-0	
Cancer (N=33)	4.62±1.23	(4.18-5.05)	2-6	<0.0001****	2.98±1.74	(2.36-3.60)	0-6	<0.0001****

CI, confidence interval; Statistical analyses were performed by Student's independent t-tests. ****p <0.0001.

**Table 3:** The CDK1 expression in nucleus and cytoplasm of normal endometrium and endometrial cancer

	CDK1 expression in nucleus				CDK1 expression in cytoplasm			
	Negative	Low	High	Low+High	Negative	Low	High	Low+High
Normal (N=30)	29 (96.7%)	1 (3.3%)	0 (0.0%)	1 (3.3%)	33 (100.0%)	0 (0.0%)	0 (0.0%)	0 (0.0%)
Cancer (N=33)	1 (3.0%)	9 (27.3%)	23 (69.7%)	32 (97.0%)	14 (42.4%)	6 (18.2%)	13 (39.4%)	19 (57.6%)

**Table 4:** CDK1 expression and clinicopathological parameters in 33 endometrial cancer patients

Parameters	CDK1 staining in nucleus			CDK1 staining in cytoplasm		
	Negative or low expression	High expression	*p* value	Negative or low expression	High expression	*p* value
**Age (years)**						
<60	3	18	0.077	12	9	0.590
≥60	5	7		8	4	
**FIGO stage**						
I	8	22	0.304	19	11	0.311
II	0	3		1	2	
**Myometrial Invasion**						
<1/2	19	11	0.783	17	10	0.9
≥1/2	1	2		2	1	
**Histological grade**						
G1	4	8	0.357	11	1	0.006**
G2	4	17		9	12	

FIGO, International Federation of Gynecology and Obsterics; Categorical variables were analyzed by the χ^2^ test. **p <0.01.
